# Dietary branched-chain amino acids intake and new-onset hypertension: a nationwide prospective cohort study in China

**DOI:** 10.1007/s00726-023-03376-0

**Published:** 2024-03-09

**Authors:** Lianlong Yu, Qianrang Zhu, Pengkun Song, Yuqian Li, Qingqing Man, Beibei Liu, Shanshan Jia, Jian Zhang

**Affiliations:** 1 Shandong Center for Disease Control and Prevention, Jinan, China; 2 National Institute for Nutrition and Health, Chinese Center for Disease Control and Prevention, Beijing, China; 3 Jiangsu Center for Disease Control and Prevention, Nanjing, China; 4 NHC Key Laboratory of Trace Element Nutrition, National Institute for Nutrition and Health, Chinese Center for Disease Control and Prevention, Beijing, 100050 China

**Keywords:** Dietary branched-chain amino acids, Hypertension, Isoleucine, Leucine, Valine

## Abstract

**Objective:**

This study aimed to investigate the relationship between dietary branched-chain amino acids (BCAAs) and the risk of developing hypertension.

**Methods:**

A cohort study of 14,883 Chinese adults without hypertension at baseline with were followed for an average of 8.9 years. Dietary intakes of BCAAs, including Ile, Leu, and Val, were collected using 3-day 24-h meal recall and household condiment weighing. Cox proportional hazards regression, restricted cubic splines, interaction analysis, and sensitivity analysis were used to assess the relationship between dietary BCAAs and risk of developing self-reported hypertension, adjusting for age, gender, region, body mass index (BMI), smoking and drinking status, physical activity, energy intake, salt intake.

**Results:**

Among 14,883 study subjects, 6386(42.9%) subjects aged ≥ 45 years at baseline, 2692 (18.1%) had new-onset hypertension during the study period, with a median age of 56 years. High levels of dietary BCAAs were associated with an increased risk of new-onset hypertension. Compared with the 41st–60th percentile, multivariable adjusted hazard ratio (HR) for new-onset hypertension was 1.16 (95% CI 1.01–1.32) for dietary BCAAs 61st–80th percentiles, 1.30 (1.13–1.50) for 81st–95th, 1.60 (1.32–1.95) for 96th–100th. The cut-off value of new-onset hypertension risk, total BCAAs, Ile, Leu, and Val were 15.7 g/day, 4.1 g/day, 6.9 g/day, 4.6 g/day, respectively, and the proportion of the population above these intake values were 13.9%, 13.1%, 15.4%, and 14.4%, respectively. Age, BMI, and salt intake had an interactive effect on this relationship (*P* < 0.001).

**Conclusion:**

There was a significant positive association between total dietary BCAAs, Ile, Leu, Val intake and the risk of developing hypertension, after adjustment for confounders. This relationship was influenced by age, BMI, and salt intake. Further research is needed to clarify the mechanism and potential role of BCAAs in the pathogenesis of hypertension.

## Introduction

Hypertension is one of the most important cause of global disease burden and death, and it is estimated that hypertension accounts for about 13% of global deaths (Leiba et al. 2019; Poulter et al. 2015) Meanwhile, hypertension is a major risk factor for cardiovascular diseases such as stroke and heart disease (Goodrich et al. 2016; Lüscher 2018; Summaries for Patients 2007). In 2016 alone, hypertension caused 10 million deaths worldwide (Kontis et al. 2019). Hypertension is a serious public health problem in any country (Yan et al. 2020). The prevalence of hypertension among Chinese adults was 27.9% (Xiang et al. 2020). Therefore, screening the risk factors of hypertension is of great significance for disease prevention.

Past literatures have demonstrated that the concentration of branched-chain amino acids (BCAAs) in the blood were positively correlated with hypertension (Yamaguchi et al. 2017; Yang et al. 2014; Mahbub et al. 2020). The accumulation of BCAA and its by-products in the body was associated with an increased risk of hypertension (Batch et al. 2013; Soleimani 2015). In addition, BCAA intake affects the synthesis of serotonin and catecholamine by inhibiting the uptake of tryptophan and tyrosine in the brain, and changes the central regulation of blood pressure (Fernstrom 2013; Choi et al. 2011). Interestingly, the hydrophobic or bulky residues in the chemical structure of BCAAs can affect the binding of angiotensin-converting enzyme to bioactive peptides, and the activity of angiotensin-converting enzyme directly affects blood pressure regulation (Martin and Deussen 2019).

However, studies on the relationship between dietary BCAAs and the risk of developing hypertension were scarce and conflicting. In a cohort study of 4288 people in an Iranian population with a 3-year follow-up, high dietary BCAAs intake and BCAA-enriched dietary patterns were positively associated with the incidence of hypertension, respectively (Teymoori et al. 2017; Mirmiran et al. 2019). While in a study involving dietary amino acid intake ratios, the ratio leucine and serine/threonine and tryptophan was significantly positively associated with the risk of hypertension (Stamler et al. 2013). However, in the Twin UK cross-sectional study involving 1997 women, dietary BCAAs were associated with a reduced risk of hypertension (Jennings et al. 2016). Moreover, there was currently a lack of data from large-scale and long-term cohort studies in this field.

The hypothesis suggests that an increment in dietary intake of branched-chain amino acids (BCAAs) may be associated with an increased risk of developing hypertension. In this study, we used a nationwide sample of Chinese adults with a mean follow-up of 8.9 years to assess the relationship between dietary BCAAs levels and the risk of new-onset hypertension. In addition, we explored thresholds of dietary BCAAs that contribute to the risk of developing new-onset hypertension.

## Methods

### Population and study design

Data for this study come from the CHNS project, an open cohort study in the Chinese population initiated by the University of North Carolina and the Chinese Center for Disease Control and Prevention (China CDC) in 1989, with follow-up every 2–4 years, and the response rate at the individual level was approximately 80% (Popkin et al. 2010). In each survey, new respondents were recruited as a supplementary sample, and by 2015, CHNS included 15 provinces/autonomous regions and 42,829 participants (Yan et al. 2022). This survey project was approved by the Institutional Review Board of the University of North Carolina at Chapel Hill and National Institute for Nutrition and Health of China CDC, and each participant signed a written informed consent. More details have been described (Popkin et al. 2010; Zhang et al. 2014). The CHNS selects study regions, communities, and households through a multi-stage random sampling process. Eligible household members are included in the study to obtain a representative understanding of the health and nutrition status of the Chinese population. The CHNS employed a systematic method for inviting participants for follow-up. After the initial baseline survey, the CHNS team maintained regular communication with the selected households. The team members visited the households periodically to collect data on health and nutrition outcomes. In addition, participants were contacted via phone calls or letters to arrange follow-up visits. This process ensured a consistent and representative sample for the duration of the study.

Since food codes in dietary data prior to 1997 were not available, seven rounds of follow-up data were used in this study in CHNS from 1997 to 2015. First, subjects who were pregnant, age less than 18 years, had a history of hypertension, or without hypertension diagnosis history, were excluded from this study. Then, subjects who participated in at least 2 rounds of survey (15,219 participants, mean follow-up 8.9 years) were included, with the first round of survey considered as baseline. The quartile range of follow-up was 1–4, 4–7, 7–14, 14–18 years. We further excluded participants with dietary energy intake > 4500 or < 600 kcal/day and who had stroke, myocardial infarction, or tumor. Finally, a total of 14,833 eligible participants were included in the study (Nansel et al. 2022; Efrati Philip et al. 2018).

### Sample size calculation validation

Based on the previous literature, the incidence of hypertension in the Chinese population was 48.6 per 1000 person years (Luo et al. 2021), and the HR of high dietary BCAAs for new-onset hypertension was 1.54 (Mirmiran et al. 2019). According to the calculation formula of the sample size of the cohort study: $n=\frac{{({{Z}_{\alpha }\sqrt{2\overline{p }\overline{q} }+Z}_{\beta }\sqrt{{p}_{0}{q}_{0}+{p}_{1}{q}_{1}})}^{2}}{{({p}_{1}-{p}_{0})}^{2}}$, with *α* = 0.05 and *β* = 0.10, the minimum sample size was 1765. This study included 14,833 subjects, and the basic needs of sample size in cohort study were being met.

### Assessment of dietary BCAAs

Dietary data for each subject in this study were assessed by a 24-h dietary recall method for 3 consecutive days and a 3-day household condiment weighing method. Three consecutive days are randomly assigned from Monday to Sunday. The accuracy of these methods for assessing dietary energy and nutrient intake has been validated (Han et al. 2020). More information on dietary measurements was available in the previous literature (Zhai et al. 2014). The 3-day 24-h dietary recall and the 3-day seasoning weight method are considered the gold standard in current dietary surveys. These methods provide reliable data by extensively documenting individuals' food intake and accurately measuring their consumption. By reducing recall bias and estimation errors, these methods offer a more authentic representation of dietary information. The fact that reputable international surveys such as NHANES employ these methods further validates their reliability and comparability. All dietary data were collected through face-to-face interviews with trained dietitians. The 3-day average intake of dietary BCAAs, energy, protein, carbohydrates, and fats at the individual level at each round of follow-up was calculated by matching the dietary data with the Chinese food composition table. Long-term dietary BCAAs exposure was measured using the mean from baseline to the last visit prior to the date of new-onset hypertension, or the mean of all visits for participants without new-onset hypertension.

### Evaluation of covariates

The main adjusted variables in the model analysis included: age, gender, region, body mass index (BMI), smoking and drinking status, physical activity, energy intake, and salt intake. Physical activity was divided into three levels of light, medium and heavy according to the questionnaire. Height and weight were measured by uniformly trained medical staff using uniformly sized equipment. BMI was calculated as weight (kg) divided by height squared (m^2^). Salt intake and BMI were averaged over several follow-up rounds, and baseline data from the first round of follow-up were used for the remaining variables.

### Results evaluation

Hypertension was based on patient-reported physician diagnosis and/or use of antihypertensive medication. Previous studies have shown that self-reported hypertension was an effective tool (Gilsanz et al. 2017). Participants had been asked to report their previous history of hypertension via a standard questionnaire at each visit. Hypertension questions in the questionnaire: (1) Has your doctor diagnosed you with hypertension? (2) How many years have you had hypertension? (3) Are you currently taking antihypertensive medication?

### Statistical analysis

Since the number of samples in this study was 14,883, the distribution of large samples can be regarded as a normal distribution according to the central limit theorem. In this study, missing data were removed and the data used were neat. For continuous variables, the means and standard deviations were reported, while proportions were reported for categorical variables. The basic information was compared using one-way ANOVA and chi-square test. Chord diagrams are used to show the food sources of the three types of BCAAs. The follow-up time for each participant was calculated from baseline to the time of diagnosis of new-onset hypertension (time of last follow-up without new-onset hypertension). Cox proportional hazards models were used to calculate hazard ratios (HR) and 95% confidence interval (CI) for the risk of new-onset hypertension with dietary BCAAs exposure, adjusting for confounding factors such as age, gender, region, BMI, smoking and drinking status, physical activity, energy intake and salt intake. Interaction analysis was used to evaluate the effect of confounding factors on the relationship between dietary BCAAs and the risk of new-onset hypertension. Restricted cubic splines (RCS) based on cox proportional hazards regression were used to explore nonlinear relationships. According to the AIC optimality principle, the RCS model with three nodes was optimal. To assess potential impact modifiers of the relationship between dietary BCAAs exposure and new-onset hypertension, further sensitivity analyses were performed. Stratified analyses were performed according to gender (male or female), age (< 45 or ≥ 45 years), BMI (< 24 or ≥ 24 kg/m^2^) (Zhou 2002), smoking status (ever or never), drinking status (yes or no), energy intake (< 2135.74 [median] or ≥ 2135.74 kcal/day) and salt intake (< 9.74 g/day [median] or ≥ 9.74 g/day), with interactions checked by likelihood ratio tests. ROC curve was used to identify cut-off values. Statistical analysis was performed using R 4.1.3 software and two-tailed *P* < 0.05 was considered statistically significant.

## Results

There were 14,883 participants included in this study. The study population was 7863 (53.0%) females, with an average age of 43.0 (14.6) years. Among the participants, smokers accounted for 31.3% (4683/14883) and drinkers accounted for 34.7% (5146/14883). Of the 14,883 subjects, 2692 (18.1%) had new-onset hypertension during the study period, with a median age of 56 years.

**Fig. 1:**
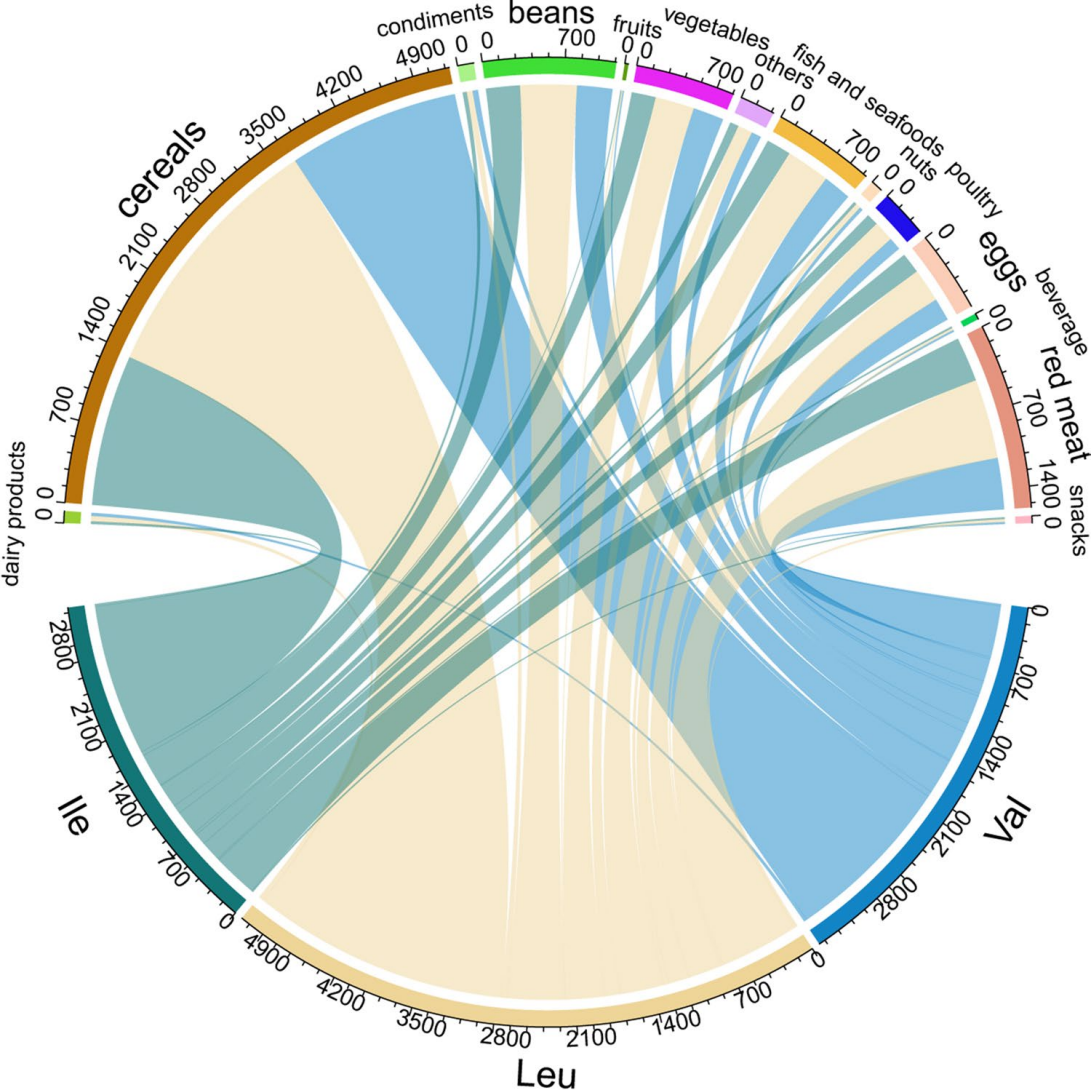
Chord diagram of food sources of three types of BCAAs. The three different colors represent the three BCAAs. The thickness of the line represents the amount of the food source

**Fig. 2:**
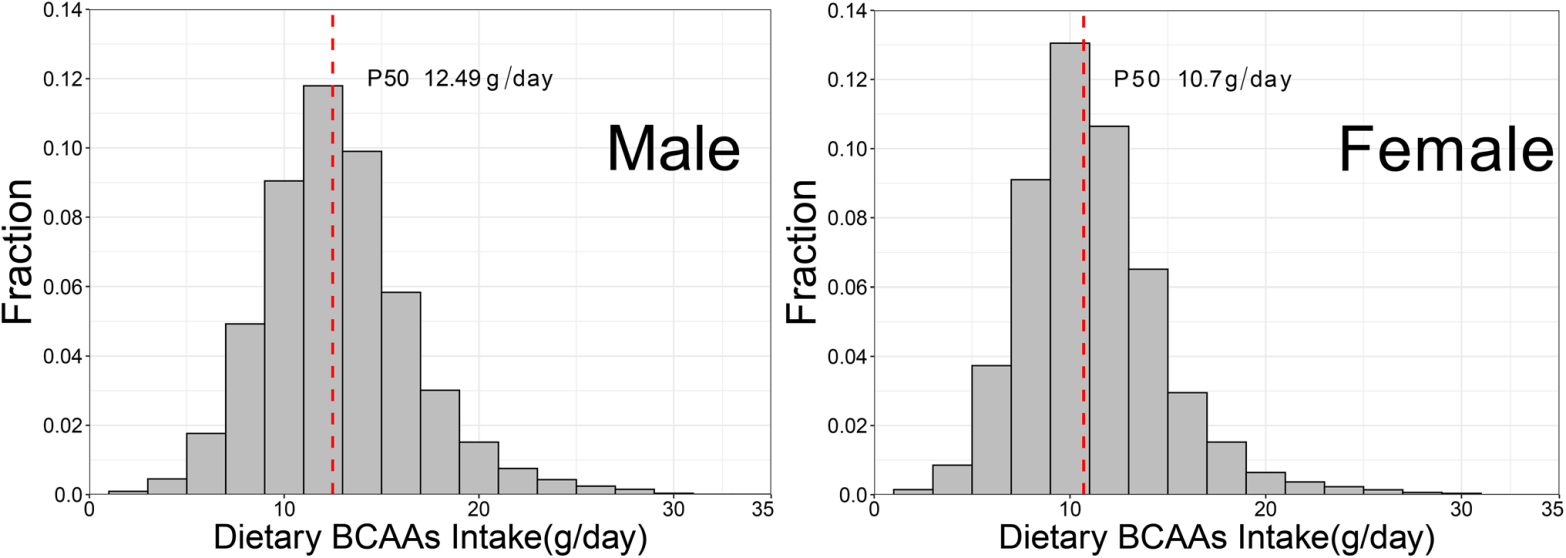
Distribution of dietary BCAAs consumption of different genders in the cohort study. The red dotted line represents the location of the median

Table 1 shows the basic characters of participants, grouped by percentile of dietary BCAAs. To explore subtle changes in the risk of new-onset hypertension caused by dietary BCAAs, we referred to validated percentile divisions (Johannesen et al. 2020). Along with the change in percentile of dietary BCAAs intake, baseline data of gender, age, smoking status, drinking status, height, weight, BMI, diastolic blood pressure, triceps skin fold, hip circumference, waist circumference, upper arm circumference, energy intake, carbohydrate intake, fat intake, protein intake were statistically different (*P* < 0.05). There was no statistical difference in systolic blood pressure among the groups (*F* = 0.39, *P* = 0.5303). The chord diagram in Fig. 1 shows that the top three main food sources of BCAAs are cereals, red meat and beans. From the histogram in Fig. 2, it can be seen intuitively that the intake of dietary BCAAs in males was greater than that in females (*t* = 27.36, *P* < 0.001).

**Table 1:** Baseline characteristics of 14,833 individuals in the CHNS Study

	All	Centile (g/day)							*F*/*χ*^2^	*P*
		1st–5th	6th–20th	21st–40th	41st–60th	61st–80th	81st–95th	96th–100th		
No of individuals	14,833	744 (5.0)	2220 (15.0)	2969 (20.0)	2964 (20.0)	2969 (20.0)	2227 (15.0)	740 (5.0)		
Women	7863 (53.0)	520 (69.9)	1511 (68.1)	1891 (63.7)	1560 (52.6)	1306 (44.0)	818 (36.7)	257 (34.7)	856.46	< 0.001
Age	43.0 (14.6)	49.8 (17.0)	47.1 (16.2)	43.4 (14.8)	41.89 (13.8)	41.23 (13.3)	40.58 (13.0)	41.51 (14.1)	78.18	< 0.001
Ever smokers	4638 (31.3)	167 (22.5)	518 (23.3)	760 (25.6)	915 (30.9)	1087 (36.6)	888 (39.9)	303 (41.0)	292.88	< 0.001
Drinker	5146 (34.7)	175 (23.5)	533 (24.0)	809 (27.3)	1021 (34.5)	1224 (41.2)	1014 (45.5)	370 (50.0)	483.38	< 0.001
Height	161.1 (8.5)	157.46 (8.3)	158.4 (8.7)	159.5 (8.2)	161.09 (8.2)	162.29 (8.0)	163.91 (8.2)	165.45 (7.8)	365.84	< 0.001
Weight	59.5 (11)	56.7 (11.2)	57.2 (11.1)	58.1 (10.6)	59.2 (10.7)	60.14 (10.3)	62.53 (11.3)	64.76 (11.5)	239.15	< 0.001
Systolic blood pressure (mm Hg)	119.0 (16.8)	124.3 (19.7)	120.7 (18.7)	118.6 (17.6)	117.53 (16.1)	117.89 (15.4)	119 (15.5)	121.01 (15.2)	0.39	0.5303
Diastolic blood pressure (mm Hg)	77.2 (10.6)	78.6 (11.3)	77.5 (11.1)	77.0 (10.8)	76.59 (10.4)	76.97 (9.9)	77.57 (10.3)	78.85 (11.1)	8.89	0.0029
Triceps skin fold (mm)	15.0 (8.0)	16.1 (8.3)	15.3 (7.9)	15.0 (8.1)	14.4 (7.9)	14.36 (8.0)	15.1 (8.0)	16.49 (8.5)	6.16	0.0131
Hip circumference (cm)	92.8 (8.4)	92.5 (8.8)	92.3 (8.4)	92.6 (8.2)	92.39 (8.3)	92.8 (8.0)	93.81 (8.2)	94.88 (9.5)	55.60	< 0.001
Waist circumference (cm)	79.7 (10.2)	79.9 (10.1)	79.4 (10.2)	79.1 (10.5)	79.04 (9.8)	79.28 (9.9)	80.87 (10.4)	82.69 (10.7)	50.24	< 0.001
Upper arm circumference (cm)	26.3 (4.1)	26.2 (4.3)	25.9 (4.2)	26.0 (3.8)	26.11 (4.0)	26.28 (4.0)	26.77 (3.9)	27.77 (5.3)	70.23	< 0.001
BMI	22.9 (3.6)	22.8 (3.8)	22.8 (4.7)	22.8 (3.3)	22.74 (3.2)	22.78 (3.2)	23.22 (3.6)	23.59 (3.4)	27.75	< 0.001
BCAAs intake (g/day)	11.9 (3.8)	5.2 (1.1)	7.8 (0.7)	9.8 (0.5)	11.52 (0.5)	13.42 (0.6)	16.21 (1.1)	21.71 (2.7)	11,062.5	< 0.001
Energy intake	2169.8 (651.0)	1292.8 (409.4)	1742.2 (480.4)	2030.1 (524.8)	2223.6 (557.1)	2375.22 (583.7)	2554.1 (621.1)	2699.56 (674.5)	1445.46	< 0.001
Carbohydrate intake	319.1 (124.5)	184.8 (71.6)	264.2 (99.2)	308.3 (107.8)	334.6 (118.1)	350.16 (124.2)	359.22 (134.6)	354.23 (136.6)	376.90	< 0.001
Fat intake	67.5 (36.0)	45.2 (29.7)	53.5 (29.8)	61.1 (32.7)	66.84 (32.8)	72.65 (35.3)	82.63 (37.6)	94 (42.3)	729.35	< 0.001
Protein intake	67.3 (22.3)	34.6 (10.3)	48.9 (10.8)	59.1 (13.1)	66.98 (15.5)	75.1 (17.1)	86.17 (19.4)	101.28 (26.2)	3861.53	< 0.001

Values are means (standard deviation) or number (%)

*BCAAs* branched-chain amino acids

The results in Table 2 showed that with increasing dietary BCAA intake, the risk of developing hypertension increased gradually. Taking the middle percentile (41st–60th) as a reference, and determining statistical significance according to the 95% CI, with the increase of dietary BCAAs intake, the 61st–80th percentile starting HR and its 95% CI have statistical significance. High levels of dietary BCAAs were associated with an increased risk of new-onset hypertension. The multivariable adjusted HR for new-onset hypertension was 1.16(95% CI 1.01–1.32) for dietary BCAAs compared with the 41st–60th percentile for 61st–80th percentiles, 1.30 (1.13–1.50) for 81st–95th, 1.60(1.32–1.95) for 96th–100th.

**Table 2:** Hazard ratios for incident hypertension according to categories of levels of BCAAs (Ile, Leu, Val)

Centile	Consumption (g/day)	Individuals	events	Event rate per 1000 person years	Model 1 Hazard ratio (95% CI)	Model 2 Hazard ratio (95% CI)
BCAAs						
1st–5th	< 6.48	744	138	36.1	0.91 (0.75–1.10)	0.98 (0.79–1.22)
6th–20th	6.48–8.85	2220	435	25.5	0.96 (0.84–1.09)	0.98 (0.85–1.14)
21st–40th	8.85–10.69	2969	525	19.1	0.99 (0.88–1.13)	1.04 (0.91–1.19)
41st–60th	10.69–12.41	2964	489	16.4	1.0	1.0
61st–80th	12.41–14.62	2969	540	18.3	1.16 (1.03–1.31)	1.16 (1.01–1.32)
81st–95th	14.62–18.73	2227	410	21.0	1.31 (1.15–1.49)	1.30 (1.13–1.50)
96th–100th	> 18.73	740	155	33.4	1.65 (1.38–1.98)	1.60 (1.32–1.95)
Ile						
1st–5th	< 1.65	742	140	37.5	0.94 (0.78–1.14)	1.04 (0.84–1.29)
6th–20th	1.65–2.28	2223	440	26.3	0.96 (0.84–1.09)	0.99 (0.85–1.14)
21st–40th	2.28–2.76	2941	529	19.4	0.98 (0.86–1.10)	1.04 (0.91–1.18)
41st–60th	2.76–3.21	2989	506	16.9	1.0	1.0
61st–80th	3.21–3.79	2986	514	17.3	1.07 (0.94–1.21)	1.09 (0.95–1.24)
81st–95th	3.79–4.82	2209	406	20.5	1.27 (1.11–1.44)	1.26 (1.09–1.45)
96th–100th	> 4.82	743	157	32.8	1.64 (1.37–1.96)	1.58 (1.30–1.92)
Leu						
1st–5th	< 2.84	742	132	34.2	0.82 (0.67–0.99)	0.90 (0.73–1.123)
6th–20th	2.84–3.90	2224	448	25.7	0.93 (0.82–1.06)	0.96 (0.84–1.11)
21st–40th	3.90–4.73	2960	501	18.0	0.91 (0.81–1.03)	0.95 (0.83–1.09)
41st–60th	4.73–5.52	2974	514	17.3	1.0	1.0
61st–80th	5.52–6.53	2966	535	18.5	1.11 (0.98–1.25)	1.11 (0.97–1.26)
81st–95th	6.53–8.41	2227	411	21.3	1.24 (1.09–1.41)	1.24 (1.08–1.43)
96th–100th	> 8.41	740	151	31.9	1.52 (1.27–1.82)	1.48 (1.21–1.80)
Val						
1st–5th	< 1.93	733	134	35.9	0.87 (0.72–1.06)	0.92 (0.74–1.15)
6th–20th	1.93–2.63	2248	444	26.2	0.93 (0.82–1.06)	0.95 (0.82–1.10)
21st–40th	2.63–3.17	2964	513	18.6	0.93 (0.82–1.05)	0.96 (0.84–1.10)
41st–60th	3.17–3.68	2972	509	17.1	1.0	1.0
61st–80th	3.68–4.32	2968	539	18.3	1.11 (0.99–1.26)	1.10 (0.96–1.25)
81st–95th	4.32–5.49	2202	401	20.5	1.26 (1.10–1.44)	1.26 (1.09–1.45)
96th–100th	> 5.49	746	152	31.6	1.57 (1.31–1.88)	1.53 (1.25–1.86)

Model 1 was adjusted for gender and age

Model 2 were adjusted for age, gender, smoking status, alcohol consumption, BMI, energy intake, salt intake, and physical activity at baseline. Based on individuals from the CHNS followed for a mean 8.9 years

**Fig. 3:**
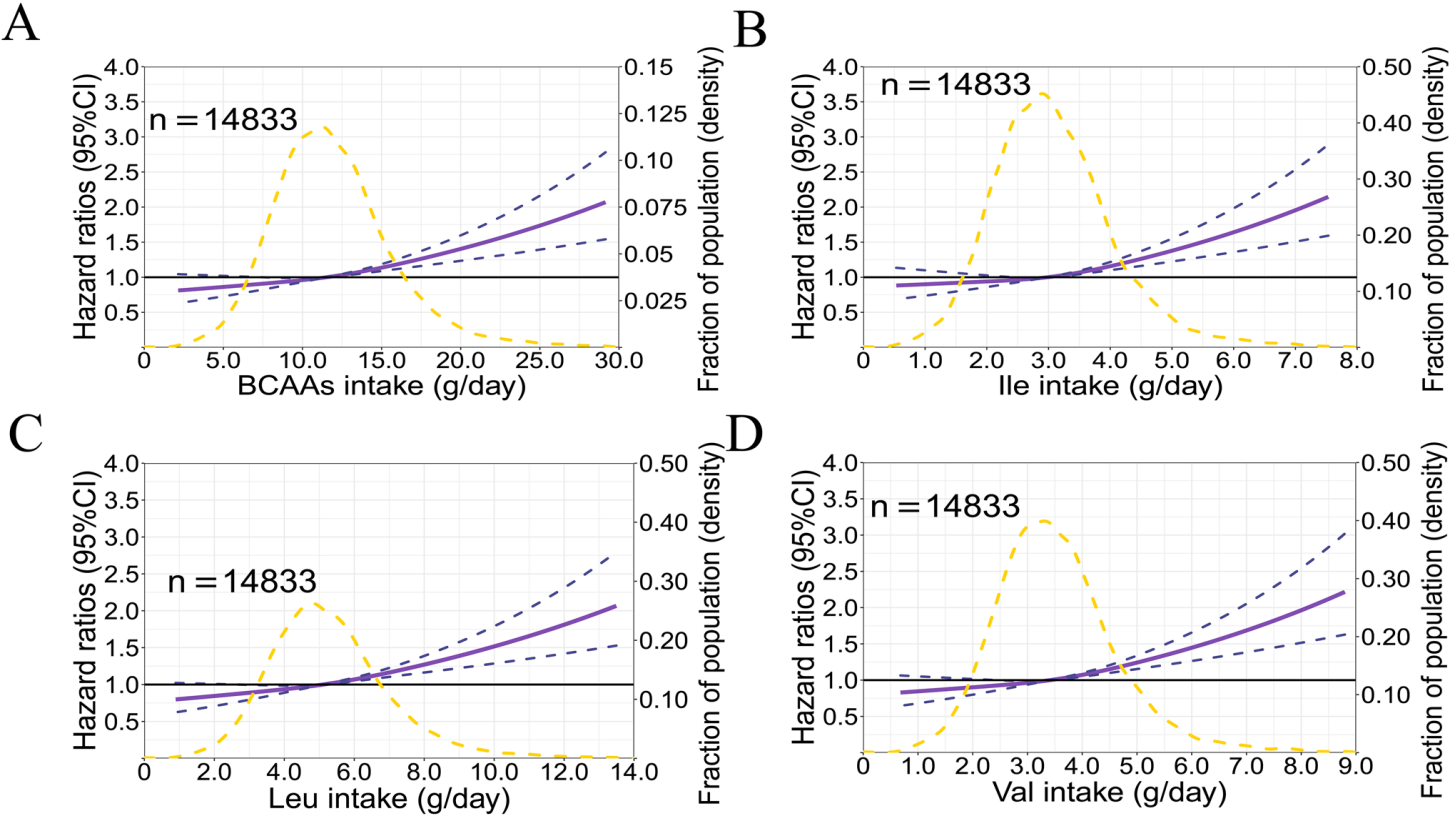
Multivariable adjusted HR of new-onset hypertension according to levels of BCAAs consumption on a continuous scale in the cohort study. Solid purple lines are multivariable adjusted HR, with dashed purples lines showing 95% CIs derived from restricted cubic spline regressions with three knots. Reference lines for no association are indicated by solid bold lines at a HR of 1.0. Dashed yellow curves show fraction of population with different levels of BCAAs intake. Analyses were adjusted for age, gender, smoking status, alcohol status, BMI and energy intake, salt intake and physical activity. Participants were followed for a mean 8.9 years in this cohort study

The HR pattern for new-onset hypertension was similar for all types of BCAAs (Ile, Leu and Val). However, for each type of BCAA, HR and 95% CI were only statistically significant above the 81st–95th percentile. Figure 3 explored HR of new-onset hypertension for dietary BCAAs using restricted cubic spline cox regression curves. Among them, the thresholds for risk occurrence were explored. The cut-off value of new-onset hypertension risk, total BCAAs, Ile, Leu, and Val were 15.7 g/day, 4.1 g/day, 6.9 g/day, 4.6 g/day, respectively, and the proportion of the population above these intake values were 13.9%, 13.1%, 15.4%, and 14.4%, respectively.

Table 3 shows the results of the stratified analysis, stratified by potential influencing factors, and found that the positive association of dietary BCAAs with the risk of new-onset hypertension was consistent in most of the stratified analyses. This association was more stable in the ≥ 45-year-old age group or the high-energy intake group. Furthermore, age, BMI and salt intake had an interactive effect on this relationship (*P* < 0.001).

**Table 3:** The association between dietary BCAAs intake and risk of new-onset hypertension in various subgroups

Subgroup	Total	New-oneset hypertension (incidence rate)	HR (95% CI)	*P*-interaction
Gender				0.1109
Female	7863	1440 (18.3)	1.298 (1.089–1.546)	
Male	6970	1252 (18.0)	1.421 (1.199–1.683)	
Age, years				< 0.001
< 45	8447	757 (9.0)	1.159 (0.905–1.484)	
≥ 45	6386	1935 (30.3)	1.41 (1.22–1.62)	
BMI				< 0.001
< 24	10,384	1441 (13.9)	1.34 (1.11–1.61)	
≥ 24	4449	1251 (28.1)	1.21 (1.02–1.43)	
Smoking status				0.7026
Never	10,195	1817 (17.8)	1.40 (1.14–1.73)	
Ever	4638	875 (18.9)	1.36 (1.17–1.58)	
Drinking status				0.6093
No	9687	1703 (17.6)	1.29 (1.06–1.56)	
Yes	5146	989 (19.2)	1.41 (1.21–1.65)	
Energy intake, kcal/day				0.3294
< 2135.74 (median)	7416	1358 (18.3)	1.12 (0.93–1.35)	
≥ 2135.74 (median)	7417	1334 (18.0)	1.36 (1.15–1.61)	
Salt intake, g/day				< 0.001
< 9.47 (median)	7143	1213 (17.0)	1.39 (1.17–1.66)	
≥ 9.47 (median)	7690	1479 (19.2)	1.32 (1.11–1.56)	

The multivariate model, if not stratified, was adjusted for age, gender, BMI, smoking and drinking status, energy intake, physical activity, and salt intake

In these models, BCAAs units were adjusted to 10 g/day

## Discussion

The top five main food sources of the three types of BCAAs were cereals, red meat, beans, fish and seafoods, vegetables. The consumption of dietary BCAAs in men was higher than that in women (*t* = 27.36, *P* < 0.001). The risk of developing hypertension was positively associated with high levels of dietary BCAAs. In the separate analysis of the three BCAAs (leucine, isoleucine, valine), the same phenomenon was observed, and the association was statistically significant (*P* < 0.05). The cut-off value of new-onset hypertension risk, total BCAAs, Ile, Leu, and Val were 15.7 g/day, 4.1 g/day, 6.9 g/day, 4.6 g/day, respectively, and the proportion of the population above these intake values were 13.9%, 13.1%, 15.4%, and 14.4%, respectively.

The research literature on the relationship between dietary BCAAs and blood pressure was very rare, and the research conclusions were inconsistent. In an Iranian cohort study of 4288 people followed for 3 years, the findings showed that higher intake of BCAAs, especially valine, was associated with higher incidence of hypertension (Teymoori et al. 2017; Mirmiran et al. 2019). In a study involving the ratio of dietary amino acid intake, the ratio of leucine and serine/threonine and tryptophan was significantly positively associated with the risk of hypertension (Stamler et al. 2013). However, in the UK twin cross-sectional study which included 1997 women, dietary BCAAs were associated with a reduced risk of hypertension (Jennings et al. 2016). In this study, higher leucine intake was inversely associated with systolic blood pressure, but not with diastolic blood pressure (Jennings et al. 2016). Conversely, in the Iranian cohort study, neither men nor women found an association between dietary leucine and the risk of new-onset hypertension (Mirmiran et al. 2019). The possible reasons for this difference are manifold and may be related to study design, race, gender, and confounding factors. In the present study, which included a larger population sample size and longer follow-up (average follow-up of 8.9 years), high dietary BCAAs intake was positively associated with the risk of new-onset hypertension, and the same phenomenons were observed in leucine, isoleucine and valine.

Recent studies have shown that BCAAs was a risk factor for many chronic diseases (Burrage et al. 2014; Magnusson et al. 2013; Newgard et al. 2009), but most of them focus on the study of BCAAs in peripheral blood (Burrage et al. 2014; Magnusson et al. 2013; Yanagisawa et al. 2015), and there are fewer studies on dietary BCAAs (Jennings et al. 2016; Nagata et al. 2013). In particular, historical literature has demonstrated that BCAA supplementation can significantly increase plasma BCAA levels (Blomstrand et al. 1996). There was a significant correlation between dietary BCAAs and plasma BCAAs levels. Eighty percent of dietary BCAAs enter the plasma, while affecting approximately 60% of valine and 50% of leucine and isoleucine plasma levels (Cavallaro et al. 2016). Studies have shown that patients with cardiovascular disease and pulmonary hypertension have significantly increased levels of BCAAs in peripheral blood (Yanagisawa et al. 2015). Increased dietary and plasma BCAAs can reduce brain serotonin, and decreasing serotonin can cause disturbances in blood pressure levels through the nervous system (Newgard et al. 2009; Wessels et al. 2016). Therefore, there was a homeostatic relationship between dietary BCAAs and plasma BCAAs. Their effects on blood pressure are inseparable from each other.

In this study, Chinese dietary BCAAs mainly came from cereals, red meat, beans, fish and seafoods, and vegetables. Previous studies have shown that high intake of whole grains and legumes were protective factors for hypertension (Liu et al. 2020; Mirizzi et al. 2019; Clark et al. 2020), and a large intake of fish can effectively reduce the incidence of hypertension (Parkhitko et al. 2020). Meanwhile, a diet rich in vegetables was the main feature of the DASH diet, which can effectively prevent and improve hypertension (Goldstein et al. 2006). However, excessive intake of red meat was a risk factor for hypertension (Schwingshackl et al. 2017; Wang et al. 2020).

In view of the fact that staple foods such as grains, legumes, and vegetables, which are the primary dietary sources of BCAAs, have been identified as protective factors against hypertension. Therefore, it can be seen that the main food source categories of BCAAs, except red meat, are not risk factors for new-onset hypertension. It was concluded that dietary BCAAs were an independent risk factor for new-onset hypertension. In the Iranian cohort study, about 57.2% of dietary BCAAs came from animal protein, which was highly correlated with animal sources (0.86). Animal protein was a confounding factor in determining relationship between dietary BCAAs and risk of new-onset hypertension. So it was difficult to adjust for animal protein in their study. In the present study, dietary BCAAs were explained to some extent as an independent risk factor for hypertension.

There were still some limitations in our study. First, we were unable to measure the correlation between dietary intake and serum BCAAs levels due to lack of data on serum amino acid concentrations. Second, although we have adjusted for potential known confounders, the effects of some residual confounding variables may still be present. Third, we acknowledge the limitation of our study in relying on self-reported hypertension, which may lead to misclassification and potentially underestimate the true prevalence. In addition, the inability to exclude all patients with hypertension at baseline was another limitation that should be noted.

The main strength of our study was the prospective cohort design, which provided some evidence of an etiological link. In addition, this was a population-based study with a large sample size and representativeness of the general population. Finally, models was adjusted for salt intake, which was a key factor affecting hypertension, and this has not been done in previous similar studies.

In conclusion, dietary BCAAs intake was greater in men than in women. There was a significant positive association between total dietary BCAAs, Ile, Leu, Val intake and risk of developing hypertension, after adjustment for confounders. Excessive intake of dietary BCAAs can be prevented through nutrition and public health communication initiatives.

## Data Availability

The data in this study are owned by the Chinese Center for Disease Control and Prevention, and data sharing is not supported at this time, but data supporting the results of this study are available from the corresponding author on reasonable request.
